# Preparation of Low-Salt-Rejection Membrane by Sodium Hypochlorite Chlorination for Concentration of Low-Concentration Magnesium Chloride Solution

**DOI:** 10.3390/ma18122824

**Published:** 2025-06-16

**Authors:** Zhengyang Wu, Zongyu Feng, Longsheng Zhao, Zheng Li, Meng Wang, Chao Xia

**Affiliations:** 1 National Engineering Research Center for Rare Earth, Grirem Advanced Materials Co., Ltd., Beijing 100088, China; wuzhengyang@grirem.com (Z.W.); lizheng@grirem.com (Z.L.); wangmeng@grirem.com (M.W.); xiachao@grirem.com (C.X.); 2 Rare Earth Functional Materials (Xiong’an) Innovation Center Co., Ltd., Xiongan 071700, China; 3 General Research Institute for Nonferrous Metals, Beijing 100088, China; 4 GRIMAT Engineering Institute Co., Ltd., Beijing 101407, China

**Keywords:** magnesium chloride solution, sodium hypochlorite, low-salt-rejection membrane, reverse osmosis, concentration

## Abstract

The precipitation process of rare earth from a rare earth chloride solution using magnesium bicarbonate yields a dilute magnesium chloride (MgCl_2_) solution. The dilute MgCl_2_ solution can only be concentrated to a maximum concentration of about 70 g/L by conventional reverse osmosis (RO), which is insufficient for recycling. Low-salt-rejection reverse osmosis (LSRRO) allows for a higher concentration of brine while operating at moderate pressures. However, research on LSRRO for the concentration of MgCl_2_ solution is still at an initial stage. In this study, polyamide RO membranes were treated with sodium hypochlorite (NaClO) to prepare low-salt-rejection membranes. The effects of NaClO concentration, pH, and chlorination time on the membrane properties were investigated. Under alkaline chlorination conditions, the membrane’s salt rejection decreased, and water flux increased with increasing NaClO concentration and chlorination time. This can be explained by the hydrolysis of polyamide in the alkaline solution to form carboxylic acids and amines, resulting in a decrease in the crosslinking degree of polyamide. The low-salt-rejection membrane was prepared by exposing it to a NaClO solution at a concentration of 15 g/L and a pH of 11 for 3 h, and the salt rejection of MgCl_2_ was 50.7%. The MgCl_2_ solution with a concentration of 20 g/L was concentrated using multi-stage LSRRO at the pressure of 5 MPa. The concentration of the concentrated brine reached 120 g/L, which is 87% higher than the theoretical maximum concentration of 64 g/L for conventional RO at the pressure of 5 MPa. The specific energy consumption (SEC) was 4.17 kWh/m^3^, which decreased by about 80% compared to that of mechanical vapor recompression (MVR). This provides an alternative route for the efficient concentration of a diluted MgCl_2_ solution with lower energy consumption.

## 1. Introduction

The conventional rare earth separation and purification process generates a large amount of salt-containing wastewater and ammonia-nitrogen wastewater with a high treatment cost. Huang’s group developed a new process of rare earth separation and purification using magnesium bicarbonate, which solves the problem of ammonia-nitrogen and high salt wastewater pollution from the source [1,2,3,4,5,6]. In this process, a magnesium bicarbonate solution is used to precipitate the rare earth chloride solution, which produces a large amount of MgCl_2_ at low concentrations (MgCl_2_ about 20 g/L). The MgCl_2_ solution is used to prepare a magnesium bicarbonate solution by alkaline conversion with calcium oxide and carbonization with carbon dioxide for magnesium bicarbonate recycling. However, in the process of alkaline conversion, if the concentration of the magnesium chloride solution is low, the obtained calcium chloride solution cannot be treated by spray-drying to produce calcium chloride products. Therefore, it is necessary to concentrate the magnesium chloride solution at a higher concentration, which can also improve the processing efficiency of the equipment and reduce investment and energy consumption in the process.

Compared with the evaporation concentration process, which needs to absorb enough heat at high temperatures to convert the liquid phase into the gas phase [7,8,9], the membrane concentration process has the advantage of operating in the liquid phase at room temperature [10,11,12]. Therefore, the membrane concentration process offers significant energy savings and has been widely used in the fields of seawater desalination and saline wastewater treatment [13,14]. It has been reported that the specific energy consumption (SEC) of the seawater reverse osmosis (RO) process is 2.5–4.0 kWh/m^3^, much less than that of mechanical vapor recompression, which is 20–25 kWh/m^3^ [15]. In the RO process, as the concentration of the brine rises, the osmotic pressure also rises, and when the concentration rises to a certain level, the osmotic pressure rises close to the operating pressure. Since the osmotic resistance is close to the hydraulic drive, it becomes difficult to further concentrate the brine. Therefore, using RO membranes to concentrate a low concentration feed solution, the brine concentration obtained is usually not more than 70 g/L, due to the limited membrane pressure [16,17,18]. It cannot fully meet the requirements for brine to reach 120 g/L for recycling.

In recent years, many emerging membrane processes have been investigated, such as forward osmosis and electrodialysis. However, their applications have been limited due to membrane cost, membrane fouling, draw solution, high energy consumption, poor perm-selectivity, and concentration polarization [19,20]. Moreover, to increase the salinity of brine without increasing the hydraulic pressure, some researchers have proposed the processes of pressure and the permeation co-driven membrane, such as low-salt-rejection reverse osmosis (LSRRO) [21], osmosis-assisted reverse osmosis (OARO) [22], and cascade osmosis-mediated reverse osmosis (COMRO) [23]. The LSRRO process requires the use of a low-salt-rejection membrane (low salt retention and high water flux) because low retention allows salt ions to pass through, thereby reducing the osmotic pressure difference between the two sides of the membrane. This reduces the resistance to water transportation at the same operating pressure, and the brine is thus further concentrated to break through the theoretical maximum brine concentration limit of conventional reverse osmosis. The multi-stage LSRRO process allows for much greater brine concentrations than the RO process, and it saves energy [24,25,26]. It is worth noting that this low-salt-rejection membrane can be produced from discarded RO membranes treated with NaClO, which significantly enhances the sustainability of the membrane-based water purification and reduces costs [27,28,29]. Low-salt-rejection membranes are characterized by their selective permeation, low-pressure operation, and easy preparation. They surpass reverse osmosis membranes in terms of energy consumption, anti-pollution, and cost, and are especially suitable for high-salt wastewater and resource recovery. However, the existing studies mainly focus on the modification of RO membranes by NaClO, without elucidating the influence mechanism of chlorination in the preparation of low-salt-rejection membranes. Moreover, the existing research on the LSRRO process is only at the theoretical and simulation stage, and there is no actual practical exploration and validation of the process [30,31]. In addition, the LSRRO process for the MgCl_2_ system has never been investigated.

In this paper, the low-salt-rejection membranes were fabricated by treating polyamide RO membranes with NaClO. The effects of the NaClO solution’s concentration, pH, and chlorination time on membrane surface composition, surface morphology, salt rejection, and water flux were investigated. The single-stage RO equipment was used to realize the multi-stage concentration process of the LSRRO to verify the feasibility and effectiveness of the LSRRO for the concentration of the low-concentration MgCl_2_ solution at the level of actual process practice for the first time.

## 2. Experimental Materials and Methods

### 2.1. Materials

Polyamide RO membrane (SW8040, Keensen, Changsha, Hunan, China) was used for this study. Sodium hypochlorite (NaClO, analytically pure) was used to configure NaClO solutions of various concentrations and pH and was purchased from Guangdong Mingde Reagent Co., Ltd., Zhaoqing, Guangdong, China. Sodium hydroxide (NaOH, analytically pure) and hydrogen chloride (HCl, analytically pure) were used to adjust the pH of NaClO solutions and were purchased from Sinopharm Chemical Reagent Co., Ltd., Shanghai, China. Magnesium chloride hexahydrate (MgCl_2_·6H_2_O, analytically pure) was used to configure the feed solution and was purchased from Xilong Science Co., Ltd., Shantou, Guangdong, China. Deionized water was prepared using the UPR-II-20L 108 ultrapure water machine form Sichuan Ulupure Ultrapure Technology Co., Ltd., Chengdu, Sichuan, China.

### 2.2. Preparation and Performance Testing of Low-Salt-Rejection Membrane

**Figure 1:**
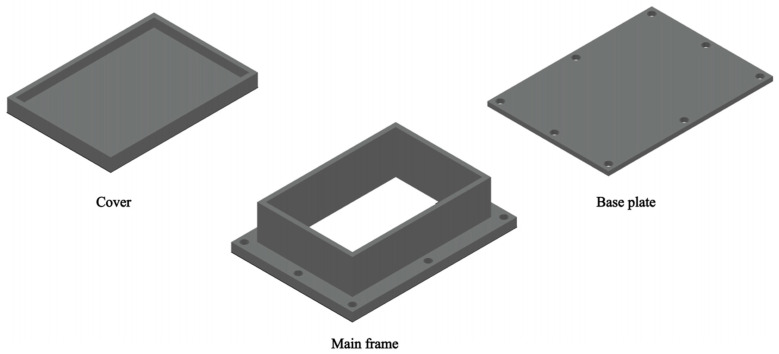
Schematic diagram of membrane preparation device.

The preparation process of the low-salt-rejection membrane was as follows: First, the membrane was placed between the main frame and the base plate of the membrane preparation device, with the polyamide active surface of the membrane facing the main frame. The schematic diagram of the device is shown in Figure 1. Then, 100 mL of the NaClO solution (0–15 g/L, pH = 5, 7, 9, or 11) was added to the device (150 mm long and 100 mm wide). The range of the NaClO solution’s concentrations chosen included a control (0 g/L), a low concentration range (0–6 g/L), and a high concentration range (8–15 g/L). A significant reduction in the salt rejection was reported at concentrations up to 15 g/L under alkaline conditions [21]. NaClO concentrations exceeding 15 g/L trigger rapid chlorination, causing immediate membrane performance loss. This is unfavorable for the controlled preparation of low-salt-rejection membranes. The pH selection included acidic, neutral, basic, and strongly basic conditions, allowing for a more comprehensive exploration of the effects of pH. The chlorination reaction between sodium hypochlorite and the polyamide active layer on the membrane surface in the device can change the membrane’s salt rejection and water flux to prepare the low-salt-rejection membrane. The device was sealed and placed in the benchtop full-temperature oscillator (TQZ-312, Jinghong, Shanghai, China) to ensure better transfer and functioning of the reactants and products within the system, resulting in a fuller reaction and a more homogeneous system. The temperature of the shaker was maintained at 25 °C and the shaker was oscillated at a speed of 50 r/min and an amplitude of 25 mm for 1–5 h. After the reaction, the solution was discarded, and 100 mL of deionized water was added to the device. The device was sealed again and shaken at a constant temperature of 25 °C for 30 min. This rinsing process was repeated three times. Finally, the prepared low-salt-rejection membranes were removed from the device, cut into three pieces of 46 mm long and 32 mm wide, and stored for future use.

**Figure 2:**
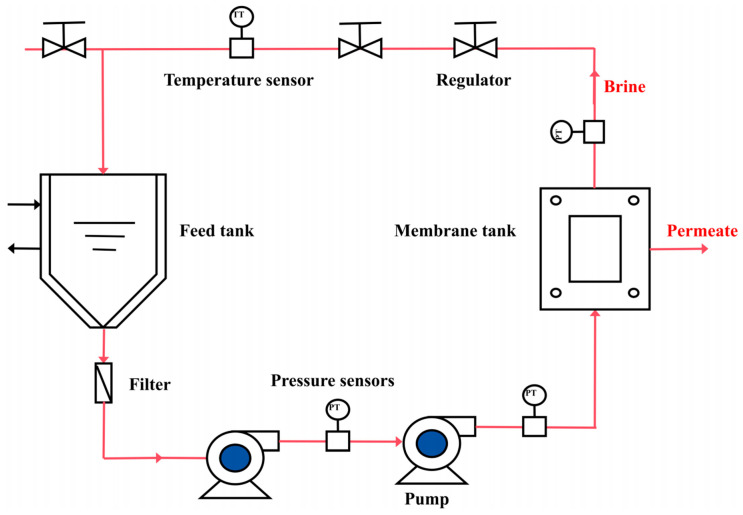
Schematic diagram of equipment flow.

The membrane performance test was conducted as follows: First, the prepared RO membrane was placed in the feed tank of the high-pressure flat-plate membrane experimental equipment and ensured to be sealed. The equipment flow chart is illustrated in Figure 2. Then, 1 L of deionized water was added to the material tank for pretreatment. The pressure was set to 1.5 MPa, and cooling water was circulated to maintain the solution temperature at 25 °C. After 1 h, the operation was stopped, and the deionized water was drained by opening the valve. Next, 1 L of the 40 g/L MgCl_2_ solution was added to the material tank. The pressure was set to 4 MPa, and cooling water was circulated to maintain the solution temperature at 25 °C. Both the concentrate and permeate were recirculated into the material tank to maintain a constant feed concentration. The operation was stopped after 2 h, and the MgCl_2_ solution was drained by opening the valve. These operations were repeated 3 times to minimize the errors. During the operation, the concentrate and permeate were collected at fixed intervals, and the volume of permeate was measured to calculate the water flux and salt rejection of the RO membrane.

The water flux is calculated as shown in Equation (1):

(1) $$J=\frac{V}{At}$$

where J is the water flux, L/(m^2^·h); V is permeate volume, L; A is the effective membrane area, m^2^; and t is the time, h.

The salt rejection of the membrane was calculated as shown in Equation (2):

(2) $$R_{T}=(1-\frac{c_{p}}{c_{f}})\times 100\%$$

where R_T_ is the salt rejection, %; c_p_ is the permeate salinity, mol/L; c_f_ is the feed salinity, mol/L.

### 2.3. Gradient Concentration Experiment

The prepared low-salt-rejection membrane was placed in the feed tank of the high-pressure flat-plate membrane experimental equipment and ensured to be properly sealed. First, 1 L of deionized water was added to the feed tank for pretreatment. The pressure was set to 1.5 MPa, and cooling water was circulated to maintain the solution temperature at 25 °C. The system was run for 1 h, after which the operation was stopped, and the deionized water was drained by opening the valve. Next, 1 L of the MgCl_2_ solution with concentrations of 40 g/L, 60 g/L, 80 g/L, or 100 g/L were sequentially added to the material tank. For each concentration, the pressure was set to 5 MPa, and cooling water was circulated to maintain the solution temperature at 25 °C. The concentrated solution was recirculated into the feed tank, while the permeate was collected using a measuring cylinder. Both the concentrate and permeate were collected at fixed intervals, and the volume of permeate was recorded. The experiment was stopped when the permeate volume reached half of the feed solution volume. Finally, the water flux and salt rejection were calculated for each concentration. These operations were repeated 3 times to minimize the errors.

### 2.4. Characterization of Membranes

The overall morphology of the membrane surface was characterized using field emission scanning electron microscopy (SEM, Phenom XL G2, Eindhoven, The Netherlands). Microscopic morphology and surface roughness were analyzed by atomic force microscopy (AFM, Bruker Icon, Saarbrucken, Germany). The chemical structure of the membrane surface was investigated using Fourier transform infrared spectrometer (ATR-FTIR, model iS50, Thermo Scientific, Waltham, MA, USA) to identify functional groups. Additionally, surface hydrophilicity was determined by measuring the water contact angle with a contact angle analyzer.

## 3. Results and Discussion

### 3.1. Infrared Spectral Analysis of Membranes

Low-salt-rejection membranes can be prepared by chlorinating polyamide RO membranes with a NaClO solution. During this process, the polyamide membranes undergo hydrolysis in the NaClO solution, and the amide bonds are cleaved, leading to changes in the membrane’s salt rejection. Furthermore, changes in the concentration, pH, and chlorination time of the NaClO solution can significantly affect the surface properties of the membrane. Changes in the chemical structure of membranes can be studied by Fourier transform infrared spectroscopy, which provides valuable insights to guide the preparation and screening of low-salt-rejection membranes.

#### 3.1.1. Effect of pH of NaClO Solution

**Figure 3:**
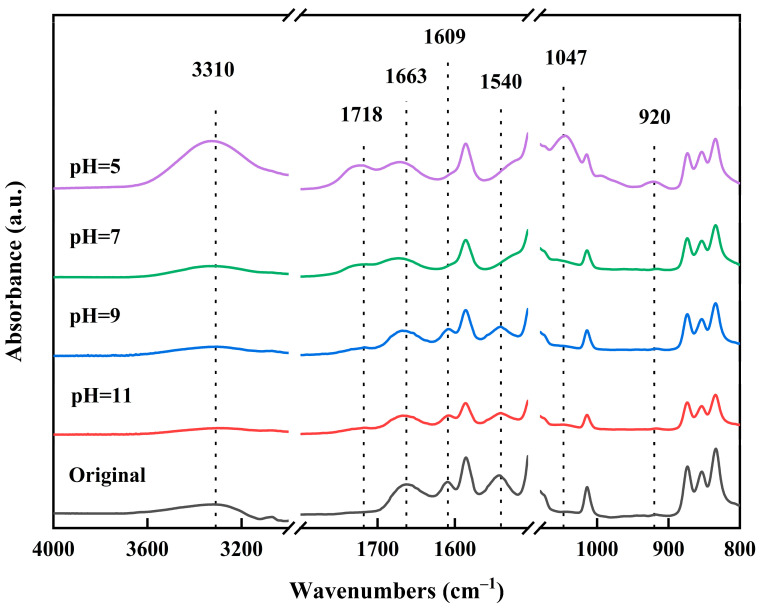
FTIR spectra of membranes treated with 2 g/L NaClO solution for 2 h at pH 5, 7, 9, 11.

The chemical structure of the polyamide active layer on the surface of the RO membrane was characterized by FTIR spectroscopy. In order to investigate the effect of pH, the chlorination conditions were set as follows: 2 g/L NaClO solution concentration; pH values of 5, 7, 9, or 11; and chlorination time of 2 h. The FTIR spectra of the polyamide membranes are shown in Figure 3.

**Figure 4:**
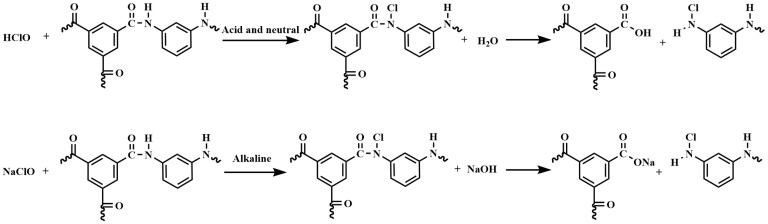
Reaction equation of chlorination.

The signature peaks of the amide functional group were clearly observed, including the amide I band (C = O stretching) at 1663 cm^−1^, the amide II band (N-H in-plane bending vibration) at 1540 cm^−1^, and the hydrogen-bonded carbonyl groups of amide at 1609 cm^−1^. Additionally, a strong and broad absorption band at 3310 cm^−1^ was assigned to both individual N-H stretching and hydrogen-bonded N-H stretching vibrations [32,33,34,35,36]. However, under acidic and neutral conditions (pH = 5 and 7), the signature peaks of the amide II band (N-H in-plane bending vibration) and the hydrogen-bonded carbonyl groups of amide disappeared, and new peaks emerged, including the C = O stretching of carboxylic acid at 1718 cm^−1^, the C-O stretching of carboxylic acid at 1047 cm^−1^, and the carboxylic acid hydrogen bonding (O-H) out-plane bending vibration at 920 cm^−1^. This is due to the fact that at low pH, highly reactive hypochlorous acid dominates the degradation process, leading to oxidative breakage of the polyamide chain to produce carboxylates (-COOH) and amines (-NH_2_), and the substitution of H atoms on the N atom by Cl atoms in partially unreacted polyamides. Under alkaline conditions (pH 9 and 11), the intensity of the amide functional group’s signature peaks decreased, and no carboxylic acid groups were observed. This is due to the fact that at high pH (alkaline), ClO- becomes more nucleophilic and preferentially generates carboxylates (-COO-) that dissolve in a solution [35], while unreacted polyamides are exposed at the surface. The hydrogen atoms in the partially unreacted polyacyl bond were rarely replaced by Cl atoms, as this was inhibited under alkaline conditions. The higher the pH, especially if it was greater than the pKa of the carboxylic acid, the more the carboxylic acid dissociated, leading to higher solubility. The more the carboxylic acid was dissolved, the faster the polyamide was hydrolyzed, resulting in a lower crosslinking degree of the membrane and a lower salt rejection. Therefore, polyamide membranes were modified with the NaClO solution under alkaline conditions to achieve low-salt rejection. Furthermore, it has been documented that chlorine, when present in an aqueous solution, undergoes a reaction with amide nitrogen, resulting in the formation of N-chlorine derivatives [36]. The reaction equation for the preparation of the low-salt-rejection membrane is shown in Figure 4.

#### 3.1.2. Effect of Concentration of NaClO Solution

**Figure 5:**
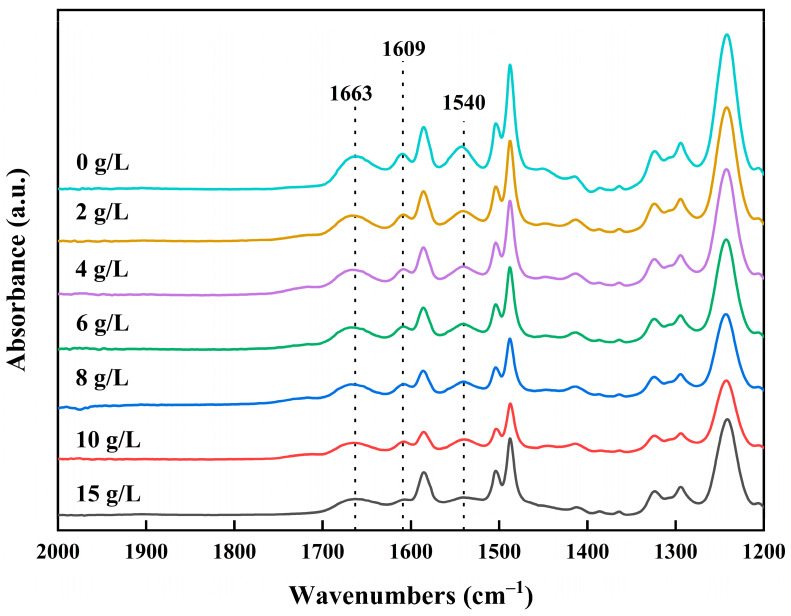
FTIR spectra of membranes treated with 0, 2, 4, 6, 8, 10, and 15 g/L NaClO solutions at pH 11 for 2 h.

It is clear that the pH of the NaClO solution has a great influence and that low-salt-rejection membranes can be prepared under alkaline conditions. Therefore, to further investigate the effect of NaClO concentration, low-salt-rejection membranes were prepared at pH 11; with NaClO solution concentrations of 2, 4, 6, 8, 10, and 15 g/L; and a chlorination time of 2 h. The FTIR spectra of the prepared low-salt-rejection membranes are shown in Figure 5. The signature peaks of the amide functional group were clearly observed, including the amide I band (C = O stretching) at 1663 cm^−1^, the amide II band (N-H in-plane bending vibration) at 1540 cm^−1^, and the hydrogen-bonded carbonyl groups of amide at 1609 cm^−1^. It was observed that the signature peaks of the amide functional groups weakened with an increasing NaClO concentration at pH 11. This proves that the higher the concentration of the NaClO solution, the more the polyamide membrane is chlorinated.

#### 3.1.3. Effect of Chlorination Time with NaClO Solution

**Figure 6:**
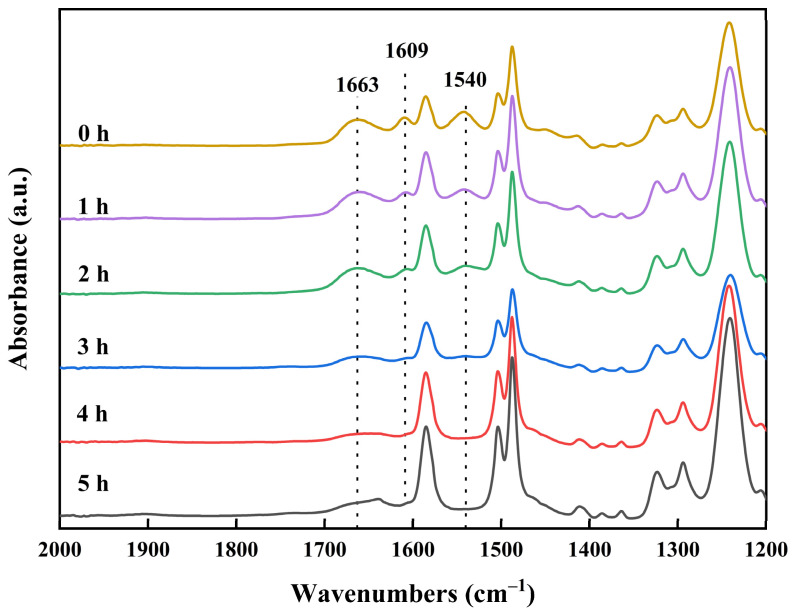
FTIR spectra of membranes treated with 15 g/L NaClO solution for 0, 1, 2, 3, 4, and 5 h at pH 11.

To precisely control the preparation of low-salt-rejection membranes and achieve controlled preparation, the effect of chlorination time must be further investigated. To clearly observe the time effect and enhance the reaction process, polyamide membranes were chlorinated using a NaClO solution at a concentration of 15 g/L and pH 11 for 0, 1, 2, 3, 4, and 5 h. However, if the concentration of the NaClO solution is greater than 15 g/L, the polyamide membrane may hydrolyze locally and rapidly, exposing the polysulfone support layer. This can lead to a rapid increase in the reaction area and rapid degradation of the polyamide active layer, which is not conducive to the controlled preparation of low-salt-rejection membranes. The FTIR spectra of the prepared low-salt-rejection membranes are shown in Figure 6. The intensity of the characteristic peaks of the amide functional group decreased as the reaction time increased. When the reaction time exceeded 4 h, the signature peaks of the amide functional group disappeared. This indicates that the polyamide was completely degraded, forming carboxylic acid and amine groups, and the carboxylic acid dissolved in water under alkaline conditions.

### 3.2. Surface Morphology Analysis of Membranes

#### 3.2.1. Effect of pH and Concentration of NaClO Solution

**Figure 7:**
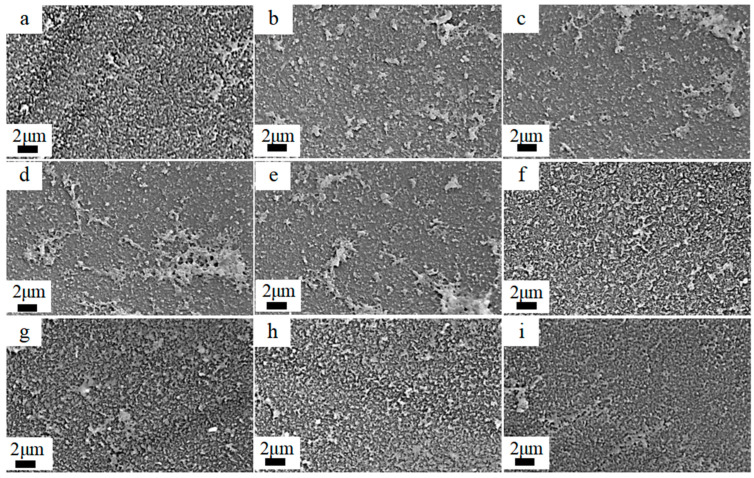
SEM plots of RO membranes prepared by 2 h treatment with different NaClO concentrations at pH 5 and 11: (**a**) pristine membrane;(**b**) 2 g/L; pH = 5, (**c**) 6 g/L; pH = 5, (**d**) 10 g/L; pH = 5, (**e**) 15 g/L; pH = 5, (**f**) 2 g/L; pH = 11, (**g**) 6 g/L; pH = 11, (**h**) 10 g/L; pH = 11, (**i**)15 g/L; pH = 11.

The surface morphology of polyamide membranes exposed to NaClO solutions under varying concentration gradients (2 h chlorination time) was characterized through scanning electron microscopy (SEM), as shown in Figure 7. The pristine membranes exhibited a distinct ridge-and-valley surface morphology. Following acidic treatment (pH = 5), membrane surfaces showed enhanced textural development, producing band-like features. Alkaline treatments (pH = 11) caused a band-like feature reduction, transitioning to discrete dot-like structures. The FTIR analysis elucidated the chemical basis of observed morphological evolution. Under acidic conditions (pH = 5), NaClO-induced polyamide hydrolysis generated amine and carboxylic acid groups, increasing the crosslinking density through hydrogen bonding and molecular packing. These charged moieties also promoted electrostatic repulsion within the polyamide, driving ridge-and-valley topography amplification. Conversely, alkaline treatment (pH = 11) caused gradual smoothing of the ridge-and-valley topography. The observed phenomenon arises from the hydrolysis of the polyamide, which liberates carboxylic acid groups that undergo alkaline dissociation, leading to the progressive dissolution of the polyamide layer. Theoretically, an increase and decrease in membrane surface ridge-and-valley topography is accompanied by an increase and decrease in membrane densification. Therefore, the reduction of ridge-and-valley topography under alkaline conditions and the reduction of membrane densities may result in lower membrane salt rejection and higher water fluxes.

In addition, the morphology of the membranes did not change significantly with the increasing NaClO concentration. This indicates that the concentration of NaClO was not the main factor affecting the morphology.

#### 3.2.2. Effect of Chlorination Time with NaClO Solution

**Figure 8:**
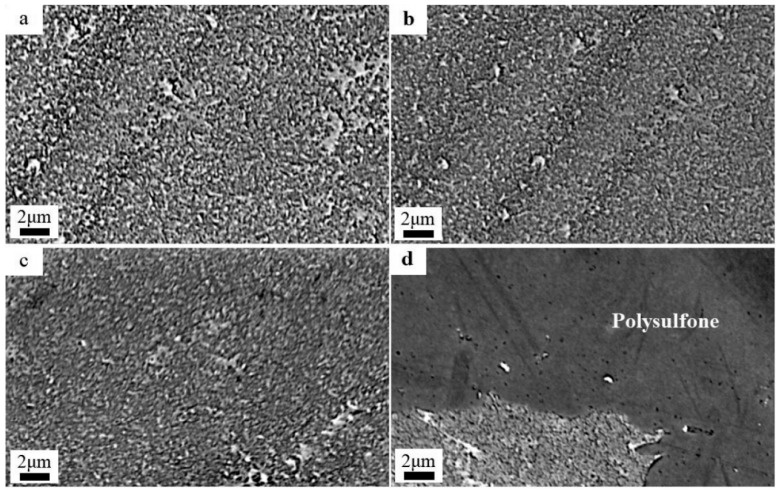
SEM plots of low-salt-rejection membranes treated with 15 g/L NaClO solution at pH 11 for different time periods: (**a**) 0 h, (**b**) 2 h, (**c**) 3 h, (**d**) 4 h.

The SEM diagrams of the RO membrane treated with 15 g/L NaClO at pH 11 under varying chlorination times are shown in Figure 8. With the increase in chlorination time, the ridge-and-valley topography of the polyamide active layer progressively attenuated, and the surface roughness decreased. This surface smoothing originated from base-catalyzed hydrolysis of the polyamide active layer, which generated carboxylic acid and amine groups. Under alkaline conditions, the resulting carboxylate anions exhibited enhanced solubility, leading to polyamide dissolution and reduced crosslinking density. When the time reached 4 h, most of the polyamide active layer had been degraded, exposing the polysulfone support layer, as shown in Figure 8d. The hydrolysis demonstrated strong time dependence, which led to a systematic reduction in crosslinking density, accompanied by surface roughness attenuation. Upon reaching 4 h exposure, almost all of the polyamide active layer had undergone degradation, fully exposing the underlying polysulfone support layer’s macrovoid structure.

**Figure 9:**
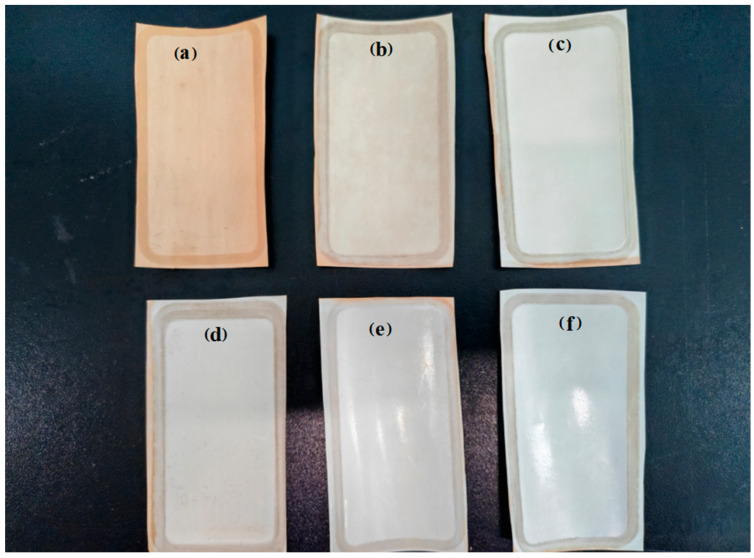
Low-salt-rejection membranes treated with 15 g/L NaClO solution at pH 11 for different time periods: (**a**) 0 h, (**b**) 1 h, (**c**) 2 h, (**d**) 3 h, (**e**) 4 h, (**f**) 5 h.

Moreover, this phenomenon is also clearly shown in the physical diagram as in Figure 9. To the naked eye, the membrane became lighter in color and smoother with increasing time in the NaClO solution treatment. When the chlorination time exceeded 4 h, the membrane became smooth and reflective due to the decomposition of the polyamide active layer and the exposure of the polysulfone support layer.

### 3.3. Changes in Water Flux and Salt Rejection of Membranes

#### 3.3.1. Effect of pH of NaClO Solution

**Figure 10:**
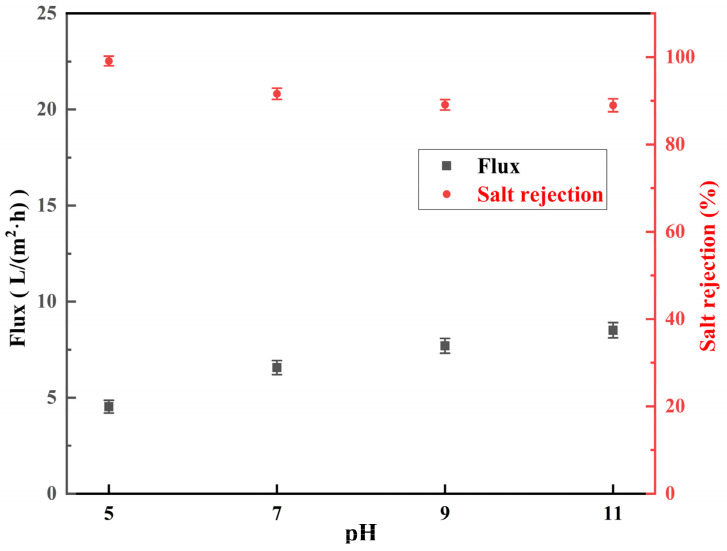
Salt rejection and water flux of RO membranes treated with 2 g/L NaClO solution for 2 h at pH 5, 7, 9, 11.

The salt rejection and water flux of low-salt-rejection membranes treated with 2 g/L NaClO at different pH for 2 h are shown in Figure 10. Under the conditions of 40g/L MgCl_2_ feed concentration and 4 MPa pressure, the pristine RO membrane was measured for baseline separation performance with a salt rejection of 94.3% ± 1% and water flux of 7.6 ± 0.3 L/(m^2^·h). At pH 5, the salt rejection of the RO membranes increased to 99% ± 1.1% and the water flux decreased to 4.5 ± 0.33 L/(m^2^·h). The salt rejections of the RO membranes treated at pH 7, 9, and 11 decreased to 91.6% ± 1.3%, 89.5% ± 1.2%, and 88.9% ± 1.5%, respectively. The water flux decreased to 5.9 ± 0.37 L/(m^2^·h) at pH 7 and increased to 7.7 ± 0.39 L/(m^2^·h) and 8.2 ± 0.4 L/(m^2^·h) at pH 9 and 11, respectively. This indicated that the salt rejection of the membranes decreased, and the water flux increased with the increasing pH of the NaClO solution. This phenomenon originates from the differential solubility behavior of carboxylic acids produced by the hydrolysis of polyamide in aqueous solutions of different pH. As the pH increased, the enhanced solubility of carboxylic acids reduced the active-layer crosslinking density of the polyamide active layer, leading to lower salt rejection and higher water fluxes. Therefore, the treatment of polyamide membranes with NaClO under alkaline conditions resulted in low-salt-rejection membranes with low salt rejection and high water fluxes.

**Figure 11:**
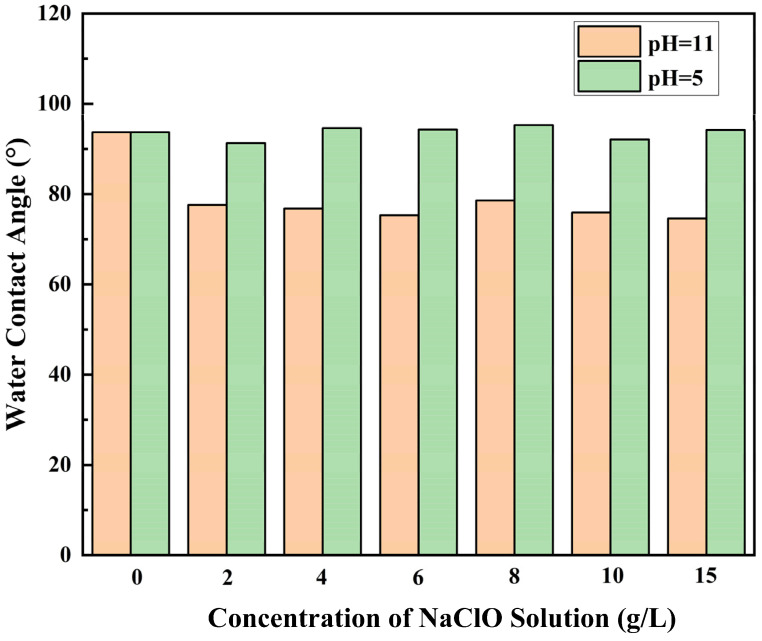
Water contact angle plots of RO membranes treated with 0, 2, 4, 6, 8, 10, and 15 g/L NaClO solutions at pH 11 and 5 for 2 h.

In addition, the surface morphology and water contact angle also affect the water flux of the membrane. The water contact angle of membranes prepared at pH 11 and 5; NaClO solution concentrations of 2, 4, 6, 8, 10, and 15 g/L; and a chlorination time of 2 h were measured in order to provide further support for the theory, as shown in Figure 11. The pristine RO membrane exhibited a water contact angle of 93 ± 0.5%, while NaClO-treated membranes showed differential behavior: acidic treatment (pH = 5) maintained a similar water contact angle (93 ± 1.5°), whereas alkaline conditions (pH = 11) significantly enhanced hydrophilicity, reducing the contact angle to 77% ± 2%, which was 17% lower than that of the pristine membrane. The alterations in the water contact angle exhibited a robust correlation with the observed morphological changes by SEM. In conditions of acidic pH, an augmentation in surface roughness was observed, concomitant with the maintenance of elevated contact angles. In contrast, the application of alkaline treatment resulted in a reduction in surface roughness, leading to the creation of smoother surfaces that facilitated enhanced water spreading. This improvement in hydrophilicity was directly associated with an increase in water flux [36], underscoring the pivotal role of surface chemistry and morphology in optimizing membrane performance.

#### 3.3.2. Effect of Concentration of NaClO Solution

**Figure 12:**
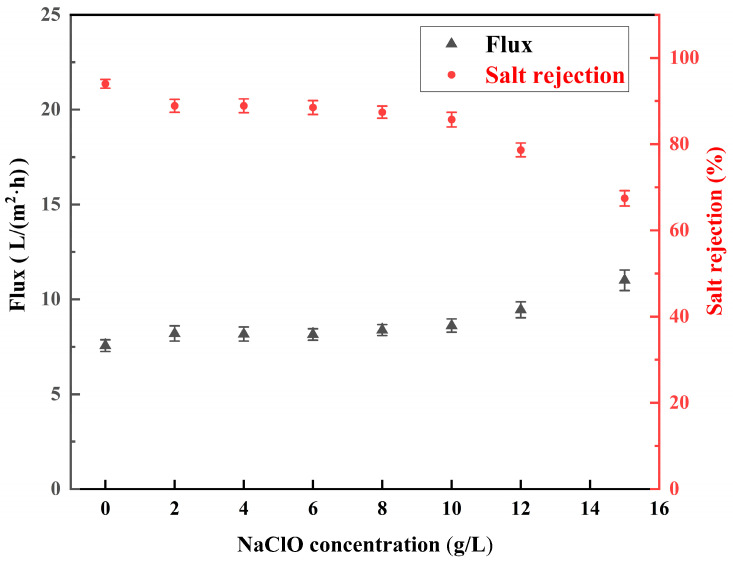
Salt rejection and water flux of low-salt-rejection membranes treated with (0–15 g/L) NaClO at pH = 11 for 2 h.

The salt rejection and water flux of the RO membranes treated with NaClO solutions (0–15 g/L) under alkaline (pH = 11) conditions for 2 h are shown in Figure 12. Under the conditions of 40g/L MgCl_2_ feed concentration and 4 MPa pressure, the pristine RO membrane was measured for baseline separation performance with a salt rejection of 94.3% ± 1% and water flux of 7.6 ± 0.3 L/(m^2^·h). The NaClO treatment of polyamide membranes could significantly reduce salt rejection and increase water flux. The salt rejection of low-salt-rejection membranes prepared by treating for 2 h at a pH of 11 and a NaClO concentration of 2 g/L was reduced to 88.9% ± 1.5%, and the water flux was increased to 8.2 ± 0.4 L/(m^2^·h). As the concentration of the NaClO solution increased, the salt rejection of the membrane slowly decreased, and then rapidly decreased when the concentration reached 10 g/L. As the concentration of the NaClO solution increased, the salt retention of the membrane was the first to decrease slowly, and then decreased rapidly when the concentration reached 10 g/L. The trend of increasing water flux was the same as the trend of decreasing salt rejection. When the concentration of NaClO reached 15 g/L, the salt rejection of the membrane was reduced to 67.4 ± 1.8% and the water flux was increased to 11.1 ± 0.54 L/(m^2^·h). This is because an increase in the concentration of the NaClO solution can enhance the chlorinated hydrolysis of polyamide, and the enhancement is more significant with a higher concentration.

#### 3.3.3. Effect of Chlorination Time with NaClO Solution

**Figure 13:**
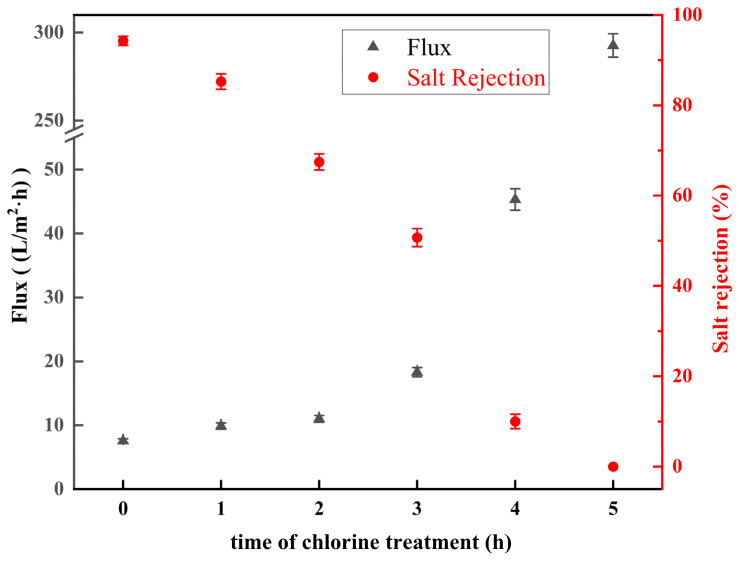
Salt rejection and water flux of low-salt-rejection membranes with 15 g/L NaClO solution at pH = 11 for 0–5 h.

In view of the limited salt-rejection reduction observed at low concentrations of NaClO, a systematic investigation was conducted into the temporal evolution of membrane performance under conditions of accelerated degradation (15 g/L NaClO, pH 11). Figure 13 presents the regulation of membrane salt rejection and water flux over various chlorination times. When the chlorination time increased from 1 h to 3 h, the salt rejection decreased to 85.3% ± 1.7%, 67.4% ± 1.8%, and 50.7% ± 2%, and the water flux increased to 9.9 ± 0.5 L/(m^2^·h), 11.1 ± 0.54 L/(m^2^·h), and 18.3 ± 0.73 L/(m^2^·h), respectively. Extended exposure to oxidative conditions resulted in a substantial decline in membrane performance. After 4 h treatment, the salt rejection plummeted to 10%±1.6%, accompanied by a water-flux escalation to 45.3 ± 1.7 L/(m^2^·h). Complete performance degradation occurred at 5 h, with salt rejection dropping to 0% and the flux reaching 278 ± 6.7 L/(m^2^·h). These structural changes were corroborated by FTIR spectroscopy, which revealed the progressive attenuation of signature polyamide absorption bands. The signature bands exhibited substantial intensity decreasing after 4 h and became undetectable following 5 h exposure. This spectral evolution confirms the time-dependent scission of amide bonds and complete degradation of the polyamide active layer under prolonged oxidative conditions. Higher NaClO concentrations (15 g/L) significantly accelerated polyamide degradation. This structural degradation triggered a critical threshold beyond which membrane performance underwent catastrophic failure: a considerable decrease in the desalination rate and a substantial increase in water flux.

### 3.4. Multi-Stage LSRRO of Low Concentration MgCl_2_ Solution

The LSRRO process employs specialized membranes with controlled selectivity to achieve target salt rejection calculated from feed and permeate concentration. According to the results in Section 3.3, the RO membrane treated under the conditions (15 g/L NaClO, pH = 11, 3 h) demonstrated modified separation characteristics. Under the testing conditions (40 g/L MgCl_2_ feed solution, 4 MPa, 25 °C), the membrane exhibited a salt rejection of 50.7% ± 2% and water flux of 18.3 ± 0.73 L/(m^2^·h). Previous studies have established that the salt rejection of a low-salt-rejection membrane increased with increasing pressure and decreased with increasing feed concentration [27]. To optimize concentration efficiency, we operated the system at 5 MPa and 25 °C, evaluating transient salt rejection across feed concentrations of 40, 60, 80, and 100 g/L MgCl_2_. The findings indicated a decline in salt rejection from 64.9% to 32.8% as the feed concentration increased from 40 to 100 g/L, a phenomenon that aligns with the principles of multi-stage LSRRO and renders it a suitable candidate for experiments. To realize real concentration processes where feed concentration increases progressively, we conducted circulation experiments with permeate discharge so that the concentration of brine could be consistently increased. RO modules have been well-studied and can easily concentrate a 20 g/L MgCl_2_ solution to 40 g/L. To achieve gradient concentration, the feed concentration was set to 40, 60, 80, and 100 g/L to investigate the LSRRO module. The pressure was set at 5 MPa, which was easy to achieve in the industry. This multi-stage LSRRO configuration continued until achieving 50% water recovery, at which point, the permeate and concentrate streams were analyzed for MgCl_2_ concentration. The measured salt rejection and water fluxes informed the design of an optimized process flow for MgCl_2_ solution concentration.

**Figure 14:**
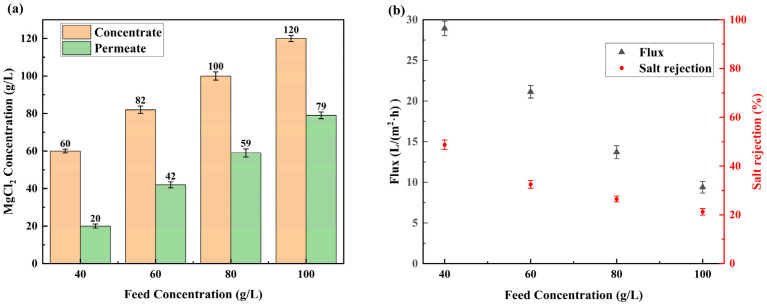
Plot of concentrate and permeate concentration, and salt rejection and water flux versus feed concentration: (**a**) concentration; (**b**) salt rejection and water flux.

The concentrations of concentrate and permeate under varying feed conditions are shown in Figure 14a. Next, 40, 60, 80, and 100 g/L of magnesium chloride solution were concentrated to about 60, 80, 100, and 120 g/L for 90, 120, 180, and 270 min, respectively, and 20, 40, 60, and 80 g/L of osmotic solution were produced, respectively. Figure 14b shows the average salt rejection and water flux of the membranes when concentrated under varying feed conditions. As the concentration of the MgCl_2_ feed solution increased from 40 g/L to 100 g/L, the water flux of the membrane decreased from 28.9 ± 0.9 L/(m^2^·h) to 9.4 ± 0.7 L/(m^2^·h), and the salt rejection of the membrane reduced from 48.7% ± 2% to 21.2% ± 1.3%. This performance degradation originated from concentration polarization effects and osmotic pressure limitations. Specifically, the elevated feed concentration increased boundary layer salt accumulation, accelerating salt transport through the membrane. Meanwhile, exponentially increasing osmotic pressure reduced the effective driving force for water permeation, as described by the solution-diffusion model. At 5 MPa pressure, when the concentration of the feed solution was greater than 120 g/L, the concentration polarization resulted in an increase in the concentration difference on the membrane surface, which led to an increase in the osmotic pressure difference close to 5 MPa, and water molecules were difficult to transport across the membrane. If the feed solution concentration was set to 120 g/L, the membrane salt rejection would drop drastically and the water flux would be reduced to an unacceptable value, leading to a significant increase in energy consumption and membrane damage.

**Figure 15:**
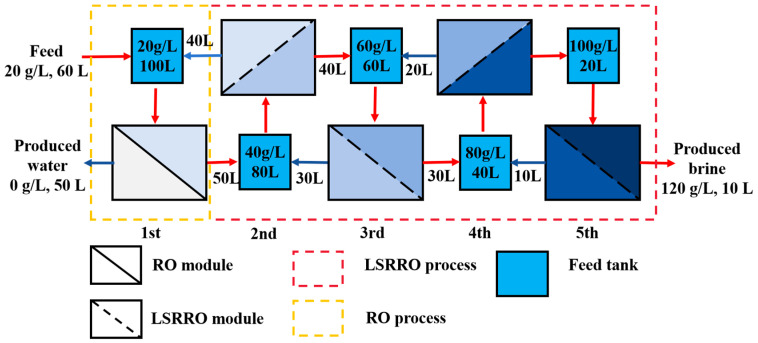
Flow chart of LSRRO at 5 MPa pressure: red arrows indicate brine flow direction, blue arrows indicate permeate return flow direction, and the color shades of the blue part in the LSRRO and RO modules represent the concentration of brine.

Through optimized stage-wise operation time control, the process can be approximated as a set of flows as shown in Figure 15 below. The first stage is the RO module, and the others are LSRRO modules. The feed solution, with a concentration of 20 g/L, first entered the RO module for concentration. This process produced a 40 g/L concentrate, which was then fed into the first-stage LSRRO module, while pure water was yielded as a permeate. The first-stage LSRRO module concentrated the 40 g/L solution to a 60 g/L concentrate that was sent to the second-stage LSRRO module, while the 20 g/L permeate was recycled back to the RO module. In each subsequent stage, the concentrate was transferred to the next stage’s feed, and the permeate was returned to the previous stage’s feed. This cycle continued until the solution reached a final concentration of 120 g/L brine.

Theoretical calculations by Van’t Hoff’s theorem showed that the theoretical limiting concentration of the RO process to concentrate the MgCl_2_ solution was about 64 g/L at 25 °C and 5 MPa [16,17,18]. The MgCl_2_ solution was successfully concentrated to 120 g/L at 5 MPa, significantly exceeding the theoretical maximum concentration limit of 64 g/L for conventional RO processes. This achievement represents an 87% increase in brine salinity compared to traditional RO treatment, demonstrating the superior concentration capability of the LSRRO.

Neglecting losses such as pump efficiency and friction, the SEC of the LSRRO process can be calculated from the following equation [21,31]:

(3) $$SEC=\frac{\sum_{i=1}^{n}Q_{P,i}\Delta P}{Q_{P}}$$

where Q_P_ is the flow rate of the produced fresh water, Q_P,i_ is the permeate flow rate in the ith stage, and ΔP is the operating pressure in the ith stage.

The SEC for concentrating a 20 g/L solution of magnesium chloride to 120 g/L at a pressure of 5 MPa was calculated as 4.17 kWh/m^3^, which is decreased by about 80% compared to that of mechanical vapor recompression (MVR). Previously reported OARO and LSRRO studies mention that the SEC obtained from computational simulations may be about 4–6 kWh/m^3^ [31]. In terms of energy consumption, the feasibility of the LSRRO process was verified with practical experiments.

### 3.5. Challenges and Future Outlook

In future work, more challenges of industrial practice factors should be taken into account, such as temperature, the real industrial raw material solution composition, and spiral-wound module, which can significantly affect the actual operational effectiveness and membrane performance of the LSRRO process. First, within a certain range, an increase in temperature will result in lower salt rejection, higher water flux, and potentially lower specific energy consumption of the membrane. However, high temperatures may lead to the destruction of the polyamide active layer. Next, the actual industry has a complex composition of solutions with impurities that can lead to varying degrees of membrane contamination and reduce membrane durability. Finally, spiral-wound membranes are commonly used in the practical industry because they have a larger membrane area and capacity compared to flat membranes used in laboratories, which reduce energy consumption. However, spiral-wound membranes are prone to clogging and (bio)contamination, which also affect the durability of the membrane. Although laboratory-scale research has been conducted, there are challenges to scaling up to the industry. At high feed concentrations, concentration polarization is non-negligible and can lead to the degradation of membrane performance and reduced concentration efficiency. Membrane fouling also has a significant impact, as it can accumulate over long periods of time, leading to reduced membrane performance.

## 4. Conclusions

The preparation of low-salt-rejection membranes and the effectiveness of the low-salt-rejection reverse osmosis process for concentrating a low-concentration magnesium chloride solution were studied and the following conclusions were obtained:

1. The reaction mechanism of the NaClO treatment on polyamide RO membranes involved the chlorination and hydrolysis of polyamide into carboxylic acid and amine groups. Since the carboxylic acid groups were insoluble in water in acidic and neutral environments, and dissolved in water in an alkaline environment, the crosslinking degree and salt rejection of the membrane were lower under the alkaline conditions. Low-salt-rejection membranes can be prepared under alkaline conditions. A higher concentration of the NaClO solution can strengthen the reaction process, and the increase in reaction time had a significant effect.
2. Under acidic conditions, the ridge-and-valley topography on the membrane surface became more pronounced, and surface roughness increased. In contrast, under alkaline conditions, the ridge-and-valley topography and surface roughness reduced, which resulted in a higher water flux. The morphology of the membranes did not change significantly with the increasing NaClO concentration. Furthermore, with a prolonged reaction time, the ridge-and-valley topography and surface roughness were further reduced. Upon reaching 4 h exposure, almost all of the polyamide active layer had undergone degradation.
3. The salt rejection of the membrane decreased with the increase in the pH, concentration, and reaction time of the NaClO solution. The water flux of the membrane increased with the increase in the pH, concentration, and reaction time of the NaClO solution. The salt rejection of the RO membrane prepared by treating for 3 h at a NaClO concentration of 15 g/L and a pH of 11 was 50.7%. Further, the salt rejection and water flux of the low desalination membrane could be regulated by changing the treatment concentration and chlorination time to realize the controllable preparation of the low desalination membrane.
4. A five-stage LSRRO process was designed based on experimental data at the level of actual process practice. The results demonstrated significant effectiveness in concentrating the MgCl_2_ solution by increasing its concentration from 20 g/L to 120 g/L under 5 MPa. This concentration was substantially higher than the theoretical maximum of 64 g/L achievable with traditional RO membranes at 5 MPa, representing an 87% improvement. The SEC for concentrating a 20 g/L solution of magnesium chloride to 120 g/L at a pressure of 5 MPa was calculated as 4.17 kWh/m^3^, which was decreased by about 80% compared to that of MVR. These findings provide evidence of the superior performance of LSRRO technology.

## Data Availability

The original contributions presented in this study are included in the article. Further inquiries can be directed to the corresponding author(s).

## References

[1] Huang X.W., Long Z.Q., Wang L.S., Feng Z.Y. (2015). Technology development for rare earth cleaner hydrometallurgy in China. Rare Met..

[2] Wu S.X., Zhao L.S., Wang L.S., Huang X.W., Zhang Y.H., Feng Z.Y., Cui D.L. (2019). Simultaneous recovery of rare earth elements and phosphorus from phosphate rock by phosphoric acid leaching and selective precipitation: Towards green process. J. Rare Earths.

[3] Li J., Li M., Zhang D., Gao K., Xu W., Wang H., Geng J., Ma X., Huang L. (2019). Clean production technology of selective decomposition of Bayan Obo rare earth concentrate by NaOH. J. Clean. Prod..

[4] Wang M., Huang X.W., Xia C., Feng Z.Y., Xu Y., Meng D.L., Peng X.L. (2023). Efficient preparation of magnesium bicarbonate from magnesium sulfate solution and saponification-extraction for rare earth separation. Trans. Nonferrous Met. Soc. China.

[5] Feng Z.Y., Huang X.W., Wang M., Zhang G.C. (2017). Progress and Trend of Green Chemistry in Extraction and Separation of Typical Rare Earth Resources. Chin. J. Rare Metals.

[6] Feng Z.Y., Wang M., Zhao L., Xu Y., Zhang Y., Huang X. (2021). Development Status and Prospect of Rare Earth Extraction and Separation Technology. J. Chin. Soc. Rare Earths.

[7] Si Z., Han D., Xiang J. (2021). Experimental investigation on the mechanical vapor recompression evaporation system coupled with multiple vacuum membrane distillation modules to treat industrial wastewater. Sep. Purif. Technol..

[8] Al-Othman A., Tawalbeh M., Assad M.E.H., Alkayyali T., Eisa A. (2018). Novel multi-stage flash (MSF) desalination plant driven by parabolic trough collectors and a solar pond: A simulation study in UAE. Desalination.

[9] Razmi A., Soltani M., Tayefeh M., Torabi M., Dusseault M.B. (2019). Thermodynamic analysis of compressed air energy storage (CAES) hybridized with a multi-effect desalination (MED) system. Energy Convers. Manag..

[10] Qasim M., Badrelzaman M., Darwish N.N., Darwish N.A., Hilal N. (2019). Reverse osmosis desalination: A state-of-the-art review. Desalination.

[11] Ali A., Tufa R.A., Macedonio F., Curcio E., Drioli E. (2018). Membrane technology in renewable-energy-driven desalination. Renew. Sustain. Energy Rev..

[12] Tang C.Y., Yang Z., Guo H., Wen J.J., Nghiem L.D., Cornelissen E. (2018). Potable Water Reuse through Advanced Membrane Technology. Environ. Sci. Technol..

[13] Goh P.S., Lau W.J., Othman M.H.D., Ismail A.F. (2018). Membrane fouling in desalination and its mitigation strategies. Desalination.

[14] Lim Y.J., Goh K., Kurihara M., Wang R. (2021). Seawater desalination by reverse osmosis: Current development and future challenges in membrane fabrication? A review. J. Membr. Sci..

[15] Kim J., Park K., Yang D.R., Hong S. (2019). A comprehensive review of energy consumption of seawater reverse osmosis desalination plants. Appl. Energy.

[16] Panagopoulos A. (2021). Techno-economic assessment of minimal liquid discharge (MLD) treatment systems for saline wastewater (brine) management and treatment. Process Saf. Environ. Prot..

[17] Davenport D.M., Deshmukh A., Werber J.R., Elimelech M. (2018). High-pressure reverse osmosis for energy-efficient hypersaline brine desalination: Current status, design considerations, and Research needs. Environ. Sci. Technol. Lett..

[18] Bartholomew T.V., Mey L., Arena J.T., Siefert N.S., Mauter M.S. (2017). Osmotically assisted reverse osmosis for high salinity brine treatment. Desalination.

[19] Ibrar I., Altaee A., Zhou J.L., Naji O., Khanafer D. (2020). Challenges and potentials of forward osmosis process in the treatment of wastewater. Crit. Rev. Environ. Sci. Technol..

[20] Al-Amshawee S., Yunus M.Y., Azoddein A.A., Hassell D.G., Dakhil I.H., Hasan H.A. (2020). Electrodialysis desalination for water and wastewater: A review. Chem. Eng. J..

[21] Wang Z., Deshmukh A., Du Y., Elimelech M. (2020). Minimal and zero liquid discharge with reverse osmosis using low-salt-rejection membranes. Water Res..

[22] Nakagawa K., Togo N., Takagi R., Shintani T., Yoshioka T., Kamio E., Matsuyama H. (2020). Multistage osmotically assisted reverse osmosis process for concentrating solutions using hollow fiber membrane modules. Chem. Eng. Res. Des..

[23] Atia A.A., Yip N.Y., Fthenakis V. (2021). Pathways for minimal and zero liquid discharge with enhanced reverse osmosis technologies: Module-scale modeling and techno-economic assessment. Desalination.

[24] Bargeman G. (2023). Maximum allowable retention for low-salt-rejection reverse osmosis membranes and its effect on concentrating undersaturated NaCl solutions to saturation. Sep. Purif. Technol..

[25] Zhao H., Wang Z., Chen Y. (2023). A theoretical analysis on upgrading desalination plants with low-salt-rejection reverse osmosis. Desalination.

[26] Du Y., Wang Z., Cooper N.J., Gilron J., Elimelech M. (2022). Module-scale analysis of low-salt-rejection reverse osmosis: Design guidelines and system performance. Water Res..

[27] Du Y., Wang L., Belgada A., Younssi S.A., Gilron J., Elimelech M. (2023). A mechanistic model for salt and water transport in leaky membranes: Implications for low-salt-rejection reverse osmosis membranes. J. Membr. Sci..

[28] Madduri S., Sodaye H.S., Debnath A.K., Adak A.K., Prasad T.L. (2023). Transformation of brackish water Reverse Osmosis membranes to nanofiltration & ultrafiltration membranes by NaOCl treatment: Kinetic and characterization studies. J. Water Process Eng..

[29] Garcia-Pacheco R., Landaburu-Aguirre J., Molina S., Rodriguez-Saez L., Teli S.B., Garcia-Calvo E. (2015). Transformation of end-of-life RO membranes into NF and UF membranes: Evaluation of membrane performance. J. Membr. Sci..

[30] Atia A.A., Allen J., Young E., Knueven B., Bartholomew T.V. (2023). Cost optimization of low-salt-rejection reverse osmosis. Desalination.

[31] Wang Z., Feng D., Chen Y., He D., Elimelech M. (2021). Comparison of Energy Consumption of Osmotically Assisted Reverse Osmosis and Low-Salt-Rejection Reverse Osmosis for Brine Management. Environ. Sci. Technol..

[32] Verbeke R., Gomez V., Koschine T., Eyley S., Szymczyk A., Dickmann M., Stimpel-Lindner T., Egger W., Thielemans W., Vankelecom I.F. (2018). Real-scale chlorination at pH4 of BW30 TFC membranes and their physicochemical characterization. J. Membr. Sci..

[33] García-Pacheco R., Landaburu-Aguirre J., Lejarazu-Larrañaga A., Rodríguez-Sáez L., Molina S., Ransome T., García-Calvo E. (2019). Free chlorine exposure dose (ppm.h) and its impact on RO membranes ageing and recycling potential. Desalination.

[34] Yang L., Yu H., Zhao H., Xia C., Yu Q., Chen X., Cao G., Cai L., Meng S., Tang C.Y. (2025). Degradation of polyamide nanofiltration membranes by free chlorine and halide ions: Kinetics, mechanisms, and implications. Water Res..

[35] Donose B.C., Sukumar S., Pidou M., Poussade Y., Keller J., Gernjak W. (2013). Effect of pH on the ageing of reverse osmosis membranes upon exposure to hypochlorite. Desalination.

[36] You M., Feng G., Fei P., Zhang Y., Cao Z., Xia J., Lau W.J., Meng J. (2021). Probing and relating the morphology, structure and performance evolution of low pressure RO membranes under chlorine exposure. J. Environ. Chem. Eng..

